# Steroid-Responsive Post-Traumatic Persistent Neutrophilic Meningitis

**DOI:** 10.1155/2022/7615939

**Published:** 2022-01-12

**Authors:** Mahboubeh Haddad, Fereshte Sheybani, Nahid Olfati, Yeganeh Azhdari

**Affiliations:** ^1^Department of Infectious Diseases and Tropical Medicine, Faculty of Medicine, Mashhad University of Medical Sciences, Mashhad, Iran; ^2^Department of Neurology, Faculty of Medicine, Mashhad University of Medical Sciences, Mashhad, Iran; ^3^Faculty of Medicine, Mashhad University of Medical Sciences, Mashhad, Iran

## Abstract

Post-traumatic meningitis is a potentially fatal condition that presents as a diagnostic and therapeutic challenge. The vast majority of post-traumatic meningitides are caused by infectious pathogens, most commonly multi-drug-resistant (MDR) bacterial pathogens. However, aseptic meningitis occurs less frequently due to tissue response to injury or stimulation by noninfectious agents, such as blood breakdown products or chemicals. Here, we present a case of post-traumatic persistent neutrophilic meningitis who was found to be steroid responsive. Diagnostic evaluation in our patient did not reveal any infectious pathogen, and the patient did not respond to broad-spectrum antimicrobial treatment. We suggest that physicians who treat patients with post-traumatic meningitis should consider steroid-responsive post-traumatic persistent neutrophilic meningitis (SPNM) in the list of differential diagnosis particularly when no infectious etiology is found and the patient does not respond to empirical antimicrobial treatment. Brain injury-induced immune dysregulation causing exaggerated inflammatory reaction might play a role in the pathogenesis of SPNM; however, further neuropathological studies are absolutely necessary to evaluate and characterize trauma-induced immune dysregulation.

## 1. Introduction

Post-traumatic meningitis is a diagnostic and therapeutic challenge. It is associated with substantial morbidity and mortality. Factors such as cerebrospinal fluid (CSF) fistula, basilar skull fracture, burr hole surgery in the emergency department, and decompressive craniectomy are associated with post-traumatic meningitis [1]. The vast majority of post-traumatic meningitides are caused by infectious pathogens, most commonly multi-drug-resistant (MDR) bacterial pathogens [2]. However, aseptic meningitis occurs less frequently due to tissue response to injury or stimulation by noninfectious agents [3]. CSF parameters can be suggestive of bacterial meningitis in the presence of compatible clinical syndrome. CSF neutrophilic pleocytosis is most often attributed to an infectious etiology; however, definitive diagnosis is confirmed when bacterial pathogens are isolated from CSF or blood specimens [4]. Here, we present a challenging clinical scenario of post-traumatic persistent neutrophilic meningitis in a young man.

## 2. Case Report

We present a 20-year-old male with a history of recent head trauma who presented to our emergency department with headache, fever, vomiting, and disorganized speech for 2 days. Head trauma had occurred about three weeks before his presentation during a motor vehicle accident leading to bilateral subdural hematoma that was managed by burr hole evacuation without craniotomy. He also developed cerebrospinal fluid (CSF) otorrhea that was managed conservatively.

**Figure 1:**
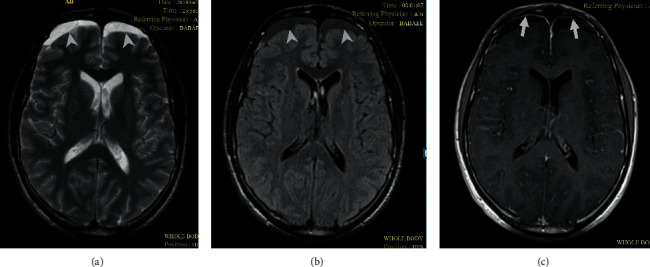
Brain magnetic resonance imaging indicative of bilateral subdural effusion (arrowheads in (a, b)) and diffuse leptomeningeal enhancement predominantly on the anterior surface of both hemispheres (arrows in (c)).

Physical examination revealed signs of meningeal irritation without focal neurological deficits. Brain magnetic resonance imaging (MRI) with contrast showed bilateral subdural effusion and diffuse leptomeningeal enhancement (Figure 1). A turbid fluid was obtained after lumbar puncture. CSF analysis revealed marked polymorphonuclear pleocytosis of 2250/*μ*L, increased protein level of 285 mg/dL, and decreased glucose level of 20 mg/dL. All microbiological tests including blood and CSF culture for rapid-growing bacteria, mycobacteria, fungi, *Nocardia* species, and *Brucella* species as well as PCR for *Mycobacterium tuberculosis* and Cryptococcal antigen repeatedly showed negative results. CSF cytological examination did not show any atypical cells.

**Figure 2:**
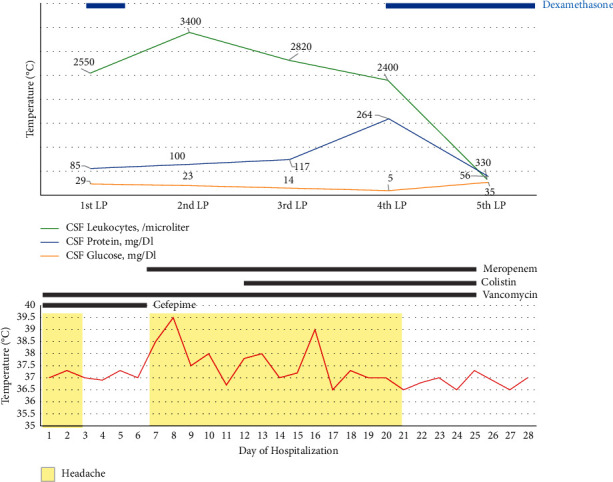
The changes of clinical and laboratory features of the patient and therapeutic regimens over time.

Broad-spectrum antimicrobial regimens did not improve clinical symptoms or CSF parameters (Figure 2). Brain MR venography, CT cisternography, and MRI of the spine did not show any additional abnormalities. There was no parameningeal source of infection that could be removed neurosurgically. Evaluation of other organ systems including echocardiography was unremarkable. Although collective clinical and paraclinical evidence was in favor of an infectious meningoencephalitis, no microorganisms were identified and response to broad-spectrum antimicrobials was disappointing.

Over the next few weeks, the patient experienced intermittent spikes of high-grade fever and rigors as well as excruciating headache and vomiting. Follow-up lumbar puncture was performed on several occasions during the first month of his hospital admission that revealed an increasing trend of CSF leukocyte count and protein levels while glucose level decreased below 20 mg/dL. Repeated evaluation of the blood and CSF for rapid-growing bacteria, mycobacteria, fungi, *Nocardia* species, and *Brucella* species revealed negative results. Review of systems and laboratory evaluations for connective tissue disorders were negative. Serum immunoglobulins and complement levels were in normal range.

On the 5th week of hospitalization, while the patient was receiving meropenem and vancomycin without any clinical response, intravenous dexamethasone at a dose of 8 mg every 8 hours was added to the therapeutic regimen which was followed by dramatic improvement in patient's clinical symptoms and signs as well as CSF parameters. Dexamethasone was tapered down gradually and discontinued after 2 months. Six and forty-month follow-up showed uneventful recovery.

## 3. Discussion

Here we presented a case of steroid-responsive post-traumatic persistent neutrophilic meningitis (SPNM). Post-traumatic meningitis is a dreadful condition that presents as a diagnostic and therapeutic challenge compared to community-acquired meningitis [5]. Diagnosing meningitis in patients with a recent neurosurgical procedure or head trauma can be challenging due to concurrent neurological deficits and/or decreased consciousness. Moreover, the typical symptoms and signs of meningeal irritation might be present in the absence of infection, mostly due to recent intracranial bleeding or neurosurgical procedures [4].

Our patient can be classified as a case of persistent neutrophilic meningitis. This syndrome is a poorly described variant of chronic meningitis characterized by the persistence of neutrophils in the CSF over a period greater than one week [6]. While bacterial, mycobacterial, and fungal pathogens are predominant causes of persistent neutrophilic meningitis particularly in cases of post-traumatic and post-neurosurgical meningitis [6–10], diagnostic evaluation in our patient did not reveal any infectious pathogen and the patient did not respond to broad-spectrum antimicrobial treatment. A history of intracranial device placement, craniotomy, or trauma resulting in contamination of the CSF and the absence of other conditions that better explain fever or altered mental status make the diagnosis of infectious meningitis more plausible [4], which firstly was the case in our patient. In addition, very high CSF neutrophil count and protein concentrations accompanied with significant hypoglycorrhachia, which were also evident in our patient, are usually best explained with an infectious etiology. Characteristic CSF pattern in bacterial meningitis has been described as glucose concentration of less than 40 mg/dL, protein concentration of over 200 mg/dL, and leukocyte count of above 1000/*μ*L with over 80% being neutrophils [11]. In fact, bacterial meningitis is highly probable (≥99% certainty) when any of the following parameters is present: a CSF glucose concentration <34 mg/dL, a protein concentration >220 mg/dL, a leukocyte count >2000/*μ*L, or a neutrophil count >1180/*μ*L [12]. However, interpretation of CSF parameters and clinical signs could be misleading in those with intracranial injuries. CSF profile can be significantly altered by intracerebral hemorrhage and/or local noninfectious inflammatory reactions [13]. This aseptic inflammation resulting from tissue response to injury or stimulation by noninfectious agents, such as blood breakdown products or chemicals, must be distinguished from infection. Although CSF culture is regarded as the gold standard for the diagnosis of infectious meningitis, 15–30% of patients with infectious meningitis have negative CSF cultures, obtained before administration of antibiotics [3].

A complete clinical and paraclinical response to steroid treatment led us to the diagnosis of steroid-responsive post-traumatic persistent neutrophilic meningitis (SPNM) in our patient. To the best of our knowledge, this is the first case of an acute post-traumatic noninfectious meningitis with severe CSF abnormalities and dramatic steroid responsiveness. Although corticosteroids are useful in improving the outcome of patients with community-acquired bacterial meningitis [14], information regarding its use in post-traumatic or post-neurosurgical meningitis is limited. It has long been recognized that acute brain injury might result in a substantial inflammatory response with peripheral and central production of proinflammatory cytokines, chemokines, and cell adhesion molecules [13]. We postulate that the meningitis syndrome in our patient was a manifestation of brain injury-induced immune dysregulation.

## 4. Conclusion

To the best of our knowledge, this is the first report of steroid-responsive post-traumatic persistent neutrophilic meningitis (SPNM) which could be added as a new clinical entity under noninfectious post-traumatic meningitides. Physicians who treat patients with post-traumatic meningitis should consider SPNM as a differential diagnosis particularly when no infectious etiologies are found and/or the patient does not respond to empirical antimicrobial treatment. Brain injury-induced immune dysregulation with exaggerated inflammatory reaction might play a role in the pathogenesis of SPNM; however, further neuropathological studies are absolutely necessary to evaluate and characterize trauma-induced immune dysregulation.

## References

[1] Katayama Y., Kitamura T., Kiyohara K. (2021). Factors associated with posttraumatic meningitis among traumatic head injury patients: a nationwide study in Japan. *European Journal of Trauma and Emergency Surgery*.

[2] Baltas I., Tsoulfa S., Sakellariou P., Vogas V., Fylaktakis M., Kondodimou A. (1994). Posttraumatic meningitis. *Neurosurgery*.

[3] Busl K. M. (2017). Nosocomial infections in the neurointensive care unit. *Neurologic Clinics*.

[4] Tunkel A. R., Hasbun R., Bhimraj A. (2017). 2017 Infectious diseases society of America’s clinical practice guidelines for healthcare-associated ventriculitis and meningitis. *Clinical Infectious Diseases*.

[5] La Russa R., Maiese A., Di Fazio N. (2020). Post-traumatic meningitis is a diagnostic challenging time: a systematic review focusing on clinical and pathological features. *International Journal of Molecular Sciences*.

[6] Peacock J. E., Mcginnis M. R., Cohen M. S. (1984). Persistent neutrophilic meningitis. *Medicine*.

[7] Green J. S., Abeles S. R., Uslan D. Z., Mehta S. R. (2011). Persistent neutrophilic meningitis in an immunocompetent patient after basilar skull fracture: case report. *BMC Infectious Diseases*.

[8] Pinto V. L., Lima M. A., Boia M. N. (2009). Persistent neutrophilic meningitis. *Journal of Neurology, Neurosurgery and Psychiatry*.

[9] Ralot T., Bafna C., Bafna C., Singh S., Sharma S. (2017). A rare presentation of tubercular meningitis as persistent neutrophilic meningitis. *Malaysian Journal of Medical Sciences*.

[10] Peacock J. E. (1990). Persistent neutrophilie meningitis. *Infectious Disease Clinics of North America*.

[11] Bijlsma M. W., Brouwer M. C., Kasanmoentalib E. S. (2016). Community-acquired bacterial meningitis in adults in the Netherlands, 2006–2014: a prospective cohort study. *The Lancet Infectious Diseases*.

[12] Spanos A., Harrell F. E., Durack D. T. (1989). Differential diagnosis of acute meningitis. *JAMA*.

[13] Caplan B., Bogner J., Brenner L., Kumar R. G., Boles J. A., Wagner A. K. (2015). Chronic inflammation after severe traumatic brain injury: characterization and associations with outcome at 6 and 12 months postinjury. *Journal of Head Trauma Rehabilitation*.

[14] Brouwer M. C., McIntyre P., Prasad K., van de Beek D. (2015). Corticosteroids for acute bacterial meningitis. *Cochrane Database System Review*.

